# Thioredoxin Modulates Cell Surface Hydrophobicity in *Acinetobacter baumannii*

**DOI:** 10.3389/fmicb.2019.02849

**Published:** 2019-12-11

**Authors:** Holly C. May, Jieh-Juen Yu, Swathi Shrihari, Janakiram Seshu, Karl E. Klose, Andrew P. Cap, James P. Chambers, M. Neal Guentzel, Bernard P. Arulanandam

**Affiliations:** ^1^Department of Biology, South Texas Center for Emerging Infectious Diseases, University of Texas at San Antonio, San Antonio, TX, United States; ^2^Acute Combat Casualty Care Research Division, U.S. Army Institute for of Surgical Research, JBSA-Fort Sam Houston, San Antonio, TX, United States

**Keywords:** *Acinetobacter*, *Francisella*, thioredoxin, hydrophobicity, type IV pili, immune evasion, virulence factor

## Abstract

*Acinetobacter baumannii*, a Gram-negative coccobacillus, has become a prevalent nosocomial health threat affecting the majority of hospitals both in the U.S. and around the globe. Microbial cell surface hydrophobicity (CSH) has previously been correlated with virulence, uptake by immune cells, and attachment to epithelial cells. A mutant strain of *A. baumannii* (ΔtrxA) lacking the redox protein thioredoxin A was found to be more hydrophobic than its wild type (WT) and complemented counterparts, as measured by both Microbial Adhesion to Hydrocarbon (MATH) and salt aggregation. The hydrophobicity of the mutant could be abrogated through treatment with sodium cyanoborohydride (SCBH). This modulation correlated with reduction of disulfide bonds, as SCBH was able to reduce 5,5′-dithio-bis-[2-nitrobenzoic acid] and treatment with the known disulfide reducer, β-mercaptoethanol, also decreased ΔtrxA CSH. Additionally, the ΔtrxA mutant was more readily taken up than WT by J774 macrophages and this differential uptake could be abrogated though SCBH treatment. When partitioned into aqueous and hydrophobic phases, ΔtrxA recovered from the hydrophobic partition was phagocytosed more readily than from the aqueous phase further supporting the contribution of CSH to *A. baumannii* uptake by phagocytes. A second Gram-negative bacterium, *Francisella novicida*, also showed the association of TrxA deficiency (FnΔtrxA) with increased hydrophobicity and uptake by J774 cells. We previously have demonstrated that modification of the type IV pilus system (T4P) was associated with the *A. baumannii* ΔtrxA phenotype, and the *Francisella* FnΔtrxA mutant also was found to have a marked T4P deficiency. Interestingly, a *F. novicida* mutant lacking pilT also showed increased hydrophobicity over FnWT. Collective evidence presented in this study suggests that Gram-negative bacterial thioredoxin mediates CSH through multiple mechanisms including disulfide-bond reduction and T4P modulation.

## Introduction

The Gram-negative multi-drug resistant (MDR) pathogen *Acinetobacter baumannii* is an important cause of nosocomial infections in the United States and worldwide (Antunes et al., 2011; Clark et al., 2016; Wieland et al., 2018). *A. baumannii* has become increasingly drug resistant, as well as possessing the ability to survive on abiotic surfaces and resist desiccation (Fournier and Richet, 2006). This led the World Health Organization to recently list the organism as the top pathogen requiring research and identification of new antimicrobials for treatment (Tacconelli and Magrini, 2017).

The role of CSH in pathogenicity and virulence has been studied previously in a number of microorganisms, where it may either promote or decrease virulence. For example, *Mycobacterium tuberculosis* has evolved to have an increasingly hydrophobic cell surface which manifests in “rough” colonies by decreasing the proportion of hydrophilic polar lipids while increasing hydrophobic non-polar lipids, which facilitated virulence and aerosol transmission, as well as rapid phagocytic uptake (Jankute et al., 2017). The Gram-positive organisms, *Staphylococcus epidermidis* and *Staphylococcus aureus*, both show a positive correlation between “interfacial free energy” (a measurement of CSH) and phagocytosis by macrophages (da Silva Domingues et al., 2013). A positive correlation was also seen in *Vibrio vulnificus* mutants with increased hydrophobicity and neutrophil phagocytosis (Kado et al., 2019). Additionally, extended spectrum beta-lactamase (ESBL)-producing *Pseudomonas aeruginosa* isolates were found to be more hydrophobic and produced increased biofilms, compared to non-ESBL counterparts, possibly rendering them more pathogenic (Norouzi et al., 2010). *Stenotrophomonas maltophilia*, a nosocomial pathogen, also showed increased hydrophobicity associated with increased adhesion to abiotic surfaces and biofilm formation with decreased swimming (flagella) motility, but not twitching motility (Pompilio et al., 2008).

In *A. baumannii*, hydrophobicity has previously been correlated with increased adherence to abiotic surfaces but not to buccal epithelial cells (Costa et al., 2006; Pour et al., 2011). Furthermore, a recent study analyzed 16 clinical isolates of *A. baumannii* in the International Clone Lineage II (IC-II) and found the more hydrophobic strains produced increased biofilms, adhered more readily to abiotic surfaces, and had decreased virulence compared to the more hydrophilic isolates (Skerniskyte et al., 2018). Our laboratory has recently suggested the previously unidentified role of thioredoxin in surface hydrophobicity of an *A. baumannii* clinical isolate Ci79 (May et al., 2019). The Ci79 strain was obtained from an US military personnel serving in Iraq (Ketter et al., 2014b). Ci79 was genome sequenced (Ketter et al., 2014a), and large-scale whole genome phylogenic analysis indicates a closer genetic relationship with AYE (IC-I) and ATCC strains 19606, 17978 than ACICU strain (IC-II). Twitching motility and pellicle formation are strongly associated with IC-I lineage strains (Eijkelkamp et al., 2011; Skerniskyte et al., 2018). Deletion of the thioredoxin A gene in Ci79 (ΔtrxA) greatly reduced twitching motility and surface type IV pili (T4P), but increased biofilm formation and cell surface hydrophobicity (CSH) as measured by Congo Red binding (May et al., 2019). Thioredoxin A (TrxA) is a small protein (12 kDa) containing a conserved active site (Cys-X-X-Cys) with a redox-active disulfate (Holmgren, 1968) and is part of the thioredoxin system, in which electrons are transferred from nicotinamide adenine dinucleotide phosphate (NADPH) to thioredoxin reductase and finally to thioredoxin. This system helps maintain a reduced intracellular compartment which assists in preventing protein aggregation (Holmgren, 1984; Stewart et al., 1998). Furthermore, TrxA from *E. coli* tagged *via* tandem affinity purification (TAP) was utilized to identify 80 TrxA associated proteins which are involved in various cellular processes such as energy transduction, transcription regulation, cell division, and several biosynthetic pathways (Kumar et al., 2004). TrxA is a ubiquitous protein present in almost all forms of life from bacteria to vertebrates. The TrxA amino acid sequences of Ci79 are identical to that of AYE, ACICU, and ATCC strains. Deletion of the *trxA* gene in Ci79 leads to attenuation in virulence in mice (Ainsworth et al., 2017; Ketter et al., 2018; May et al., 2019).

In this study, we explore the possible TrxA-dependent virulence mechanisms affecting hydrophobicity and present several lines of evidences to demonstrate that TrxA can modulate *A. baumannii* and other Gram-negative bacterial CSH through disulfide-bond reduction and regulation of type IV pili.

## Materials and Methods

### Bacterial Strains

Generation of the Ci79 ΔtrxA and complemented (Comp) strains was reported previously (Ketter et al., 2018). Unless otherwise stated, all bacteria were grown from frozen stocks on Luria-Bertani (LB) agar plates, supplemented with 50 μg/ml kanamycin to prevent contamination and, for ΔtrxA and Comp only, erythromycin for selection, and incubated at 37°C for 24 h before subculturing in LB broth. Kanamycin serves a preventive measure to minimize potential contamination throughout routine manipulations for WT Ci79, while also serving as a control to the erythromycin used in the growth of trxA mutant and complement strains. Both kanamycin and erythromycin act by inhibition of protein synthesis by binding to the ribosomal RNA. Overnight liquid bacterial cultures were diluted 1:100 and grown to mid-log phase. Growth curves for bacterial strains were generated based on optical density measured at 600 nm. Actual inoculum doses were determined by plating serial dilutions on LB agar plates. *Francisella novicida* U112 WT, FnΔtrxA was propagated from an FnU112 transposon library kindly provided by the University of Washington (Gallagher et al., 2007); the transposon insertion was verified by sequencing. FnΔpilT was generated by targeted mutagenesis in our previous study (Zogaj et al., 2008). All *F. novicida* bacterial strains were grown at 37°C in tryptic soy broth or agar (TSB or TSA, obtained from BD Biosciences, San Jose, CA) supplemented with 0.1% (w/v) L-cysteine (Fisher Scientific, Hampton, NH).

### Microbial Adhesion to Hydrocarbon Assay

The MATH assay was conducted as previously described (Rosenberg, 2006; Chao et al., 2014). In brief, bacteria were spun down (3,500 × *g* for 10 min) and washed once with PUM buffer (22.2 g K_2_HPO_4_·3H_2_O, 7.26 g KH_2_PO_4_, 1.8 g urea, 0.2 g MgSO_4_·7H_2_O in 1 L ultrapure water, pH 7.1 and sterilized by filtration using a cellulose membrane with a pore size of 0.2 μm) then resuspended in PUM buffer to an OD_600nm_ between 0.7 and 1.0 (=ODorignal). The bacteria were then mixed with 1:100 (v/v) n-hexadecane (Fisher Scientific) and vortexed vigorously for 2 min. The suspension was rested for 15 min to allow for phase separation. The aqueous partition was collected for OD_600nm_ measurement (=ODfinal). Adhesion to the hydrocarbon was measured using the following formula: %Adherence = [1 − (ODfinal/ODoriginal)] × 100.

### Salt Aggregation Test

The Salt Aggregation Test (SAT) was performed as described previously (Lindahl et al., 1981; Skerniskyte et al., 2018). Briefly, *A. baumannii* were grown overnight on LB agar plates at 37°C. Bacterial colonies were resuspended with ddH_2_O and adjusted to an OD_600nm_ between 0.7 and 1.0. Twenty-five microliter of the bacterial suspension was mixed with 25 μl of ammonium sulfate of varying molarity (0.2–2 M in increments of 0.2). For aide of visualization, 40 μl of 0.1% methylene blue was added to each 1 ml aliquot of ammonium sulfate. The mixture was gently rocked for 5 min at 25°C before visualization at 40× magnification. Results are recorded as the lowest salt concentration in which the bacteria showed aggregation.

### Disulfide Reduction Assay

The DTNB reduction assay was conducted as described previously (Ketter et al., 2018). Briefly, bacteria were grown at 37°C to an OD_600nm_ of 0.7. Each strain was pelleted at 3,000 × *g* and washed three times in M9MM (48 mM Na_2_HPO_4_, 167 mM KH_2_PO_4_, 8.5 mM NaCl, 19 mM NH_4_Cl, 2 mM MgSO_4_, 100 μM CaCl_2_, 0.4% glucose). Bacterial pellets were suspended in either sterile M9MM alone or M9MM supplemented with 1 mM DTNB and grown for 24 h at 37°C, at which point supernatants were collected. For testing of redox properties of SCBH, a concentration of 2.0 mg/ml was added to the M9MM with or without DTNB. Reducing activity was observed through yellow color development, i.e., the reduction of DTNB (colorless) to TNB (bright yellow), detected at 450 nm using a Spark 10 M Microplate reader (Tecan, Zürich, Switzerland).

### Chemical Modulation of Hydrophobicity

Bacteria in mid-log phase were spun down (3,500 × *g* for 10 min), washed once in PUM buffer, and resuspended in 50 mM sodium acetate buffer (pH 4.5) or a sodium acetate buffer containing 2.0 mg/ml of sodium cyanoborohydride (SCBH) and placed overnight in 4°C. Bacteria were then washed twice with either PUM buffer or PBS before experimental testing. For bacterial growth in the presence of SCBH, bacteria were plated on a 96-well plate in LB with or without 2.0 mg/ml of SCBH. The OD_600_ was measured every 2 for 8 h and additionally at 12 and 24 h.

Hydrophobicity also was modulated with β-mercaptoethanol treatment. Bacteria in mid-log phase were spun down (3,500 × *g* for 10 min) and resuspended in PUM buffer. Bacteria were then incubated with β-mercaptoethanol (20% v/v in PBS) at a 1:1 ratio at room temperature for 10 min, then spun down, washed once, and resuspended in PUM buffer for downstream experiments.

### Bacterial Uptake by J774 Cells

J774 macrophage-like cells were seeded into 96-well plates at a density of 5 × 10^4^ cells/ml in DMEM supplemented with 10% fetal bovine serum and placed overnight in a 37°C incubator with 5% CO_2_. Four hours prior to infection, J774 cells were activated with 10 ng/ml of LPS (Bartosh and Ylostalo, 2014) and then infected with 5 × 10^6^ CFU of specified bacteria. One hour later, cells were washed twice with DMEM and treated with either 50 μg/ml polymyxin (*A. baumannii*) or 20 μg/ml gentamicin (*F. novicida*) for 2 h to kill extracellular bacteria. Cells were washed twice with DMEM then lysed with 0.1% Triton-100. Bacterial CFUs were enumerated using standard dilution plate counting.

### Transmission Electron Microscopy

*F. novicida* was grown for 16 h with shaking (100 rpm) at 37°C, collected by centrifugation (3,000 × *g* for 10 min), and resuspended in PBS. Bacteria were placed onto a Formvar/carbon coated grid, stained with 2% (w/v) uranyl acetate, and examined using a JEM-1400(Plus) Transmission Electron Microscope (Electron Microscopy Lab, The University of Texas Health Science Center at San Antonio).

### Statistics

Statistical differences were assessed by ANOVA with Holm-Sidak correction for multiple comparisons. Statistics were conducted using GraphPad Prism statistical software (San Diego, California).

## Results

### Loss of Thioredoxin Leads to Changes in Cell Surface Hydrophobicity

**Figure 1:**
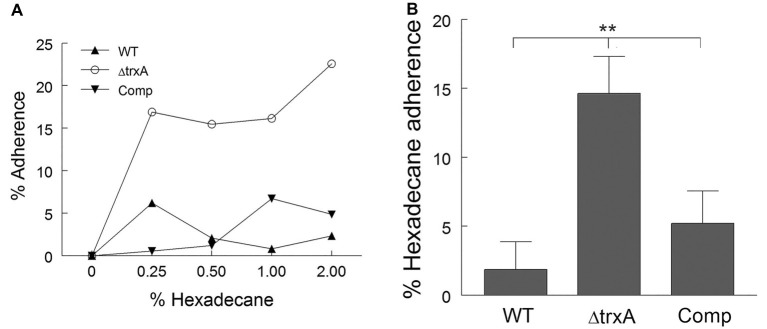
Loss of thioredoxin increases cell surface hydrophobicity. **(A)** Cell surface hydrophobicity of wild type (WT), ΔtrxA and complement (Comp) strains was measured using Microbial Adhesion to Hydrocarbon, with various concentrations of n-hexadecane. **(B)** Adherence to n-hexadecane at 1:100 concentration of n-hexadecane to bacterial suspension. Statistical differences were determined by one-way ANOVA with Holm-Sidak correction for multiple comparisons (^**^p < 0.01).

Previously, our laboratory noted the differential binding of Congo Red dye to the cell wall of the WT *A. baumannii* and a ΔtrxA mutant (May et al., 2019) which prompted further exploration into possible modulation of cell surface hydrophobicity by thioredoxin. Using the microbial adhesion to hydrocarbon (MATH) assay, a well-accepted hydrophobicity measurement (Rosenberg, 2006; Zoueki et al., 2010; Kado et al., 2019), the cell surface hydrophobicity of WT and ΔtrxA was assessed with the assumption that more hydrophobic bacteria will adhere more readily to the hydrocarbon than hydrophilic bacteria. Various dilutions of n-hexadecane were tested (Figure 1A), and 1:100 was chosen for the remainder of this study. The WT and complemented (Comp) strain showed approximately 2 and 5% adherence to the hydrocarbon while the ΔtrxA mutant was approximately 15% (Figure 1B). To further support this TrxA-associated differential hydrophobicity, the salt aggregation test (SAT) was conducted as an additional method to measure bacterial hydrophobicity. We observed that ΔtrxA formed bacterial aggregates at a molarity of 0.6 while both WT and Comp aggregated at a much higher salt concentration of 1 M. Collective results from Congo Red binding, MATH and SAT indicated that deletion of the *trxA* gene in *A. baumannii* resulted in an increased bacterial surface hydrophobicity.

### Cell Surface Hydrophobicity Can Be Modulated With TrxA-Like Thio-reduction

**Figure 2:**
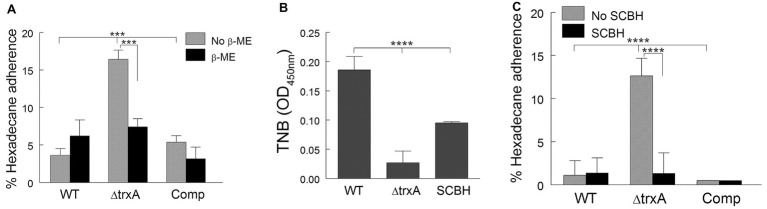
Modulation of cell surface hydrophobicity is dependent on disulfide bond reduction. **(A)** Adherence of wild type (WT), ΔtrxA and complement (Comp) strains to n-hexadecane with and without β-mercaptoethanol (β-ME) treatment. **(B)** Reduction of disulfide bonds by SCBH as measured through reduction of DTNB to TNB. **(C)** Adherence to n-hexadecane with and without sodium cyanoborohydride (SCBH) treatment. Statistical differences were determined by one-way **(B)** or two-way **(A,C)** ANOVA with Holm-Sidak correction for multiple comparisons (^****^p < 0.0001, ^***^p < 0.001).

Thioredoxins are efficient disulfide reductants due to their low redox-potential (Krause et al., 1991). To investigate whether the increase of CSH in ΔtrxA is associated with decreased reducing capacity, we treated WT, mutant, and Comp with β-mercaptoethanol, a well-known disulfide bond reducer. As shown in Figure 2A, when treated with β-mercaptoethanol, the level of ΔtrxA adherence to n-hexadecane was reduced to levels similar to that of both the treated and non-treated WT and TrxA Comp strains suggesting the contribution of thio-reduction to TrxA-modulated CSH. To confirm this finding, we used a second reducing agent, sodium cyanoborohydride (SCBH). As previously documented (Ketter et al., 2018), the ΔtrxA mutant is unable to reduce the membrane-impermeable 5,5′-dithio-bis-[2-nitrobenzoic acid] (DTNB) at similar levels to the WT. In comparison, SCBH (2.0 mg/ml) alone was able to reduce DTNB significantly more than ΔtrxA (Figure 2B). Additionally, incubation of ΔtrxA with SCBH markedly reduced hydrophobicity, as measured by MATH, to a level comparable to WT and Comp strains (Figure 2C). No further reduction in the already low CSH was seen when WT and Comp were treated with SCBH. To the best of our knowledge, this is the first study showing SCBH can be used to modulate CSH.

### Cell Surface Hydrophobicity Affects Phagocytosis

**Figure 3:**
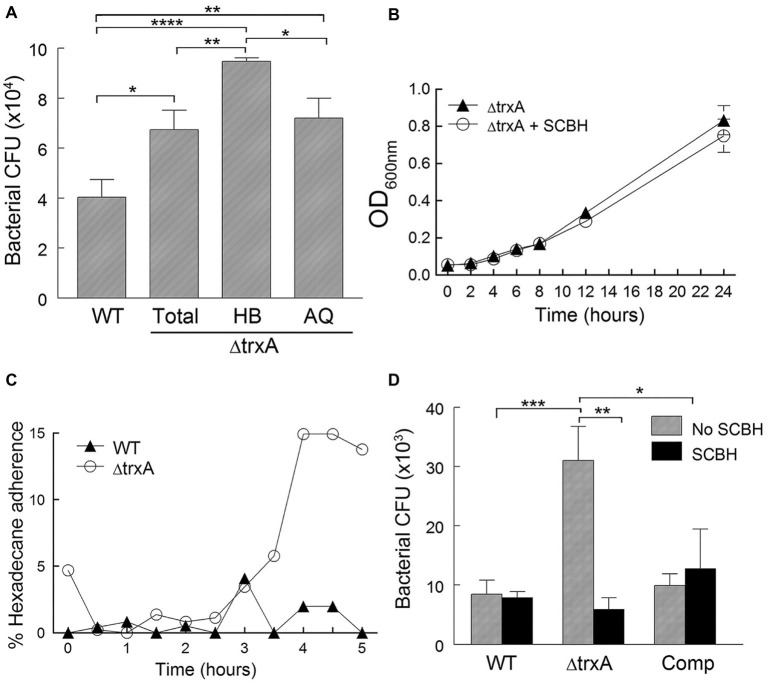
Bacterial cell surface hydrophobicity modulates phagocytosis. **(A)** Recovered CFU/well of bacteria phagocytosed by J774 cells from WT, ΔtrxA (Total), ΔtrxA (HB) hydrophobic partition, and ΔtrxA (AQ) aqueous partition. **(B)** Bacterial growth in the presence of sodium cyanoborohydride (SCBH). **(C)** Time course of the retention of bacterial hydrophilicity after SCBH treatment. **(D)** Recovered CFU/well of bacteria, with or without SCBH treatment, following phagocytosis (1-h bacterial uptake followed by 2-h antibiotic treatment) by J774 cells. Statistical differences were determined by one-way **(A)** and two-way **(D)** ANOVA with Holm-Sidak correction for multiple comparisons (^****^p < 0.0001; ^***^p < 0.001; ^**^p < 0.01; ^*^p < 0.05).

The thioredoxin system has been shown to play an important role in various cellular functions and in host immune evasion. We set forth to assess whether bacterial TrxA plays a role in the evasion of phagocytic uptake of *A. baumannii via* modulation of CSH. As shown in Figure 3A, ΔtrxA (total population) was phagocytosed more readily by macrophage-like J774 cells, after an hour-long incubation, than WT. The ΔtrxA mutant was then partitioned with n-hexadecane into more hydrophobic (HB) and more hydrophilic (AQ) fractions. The J774 cells took up the HB phase more readily than the AQ phase of ΔtrxA, suggesting the association of hydrophobicity with loss of thioredoxin in TrxA-dependent evasion of phagocytosis (Figure 3A). To further test this hypothesis, ΔtrxA reduced by chemical treatment was used to observe the effect on bacterial uptake by J774 cells. The effects of SCBH treatment on the ΔtrxA mutant were measured in two ways: (1) growth of the SCBH treated and untreated mutant over a 24-h period were similar (Figure 3B), and (2) the treated mutant retained low surface hydrophobicity for 3 h after removing SCBH from the bacterial culture (Figure 3C). These two critical properties allowed us to investigate the contribution of CSH to TrxA mediated evasion from phagocytes using SCBH treated ΔtrxA. As shown in Figure 3D, the normal increase in ΔtrxA mutant uptake by J774 cells, compared with WT and Comp strains, was abrogated by treatment with SCBH supporting our hypothesis that *A. baumannii* can evade immune cell phagocytosis by TrxA-mediated modulation of surface hydrophobicity.

### TrxA Modulates CSH in Other Gram-negative Bacteria

**Figure 4:**
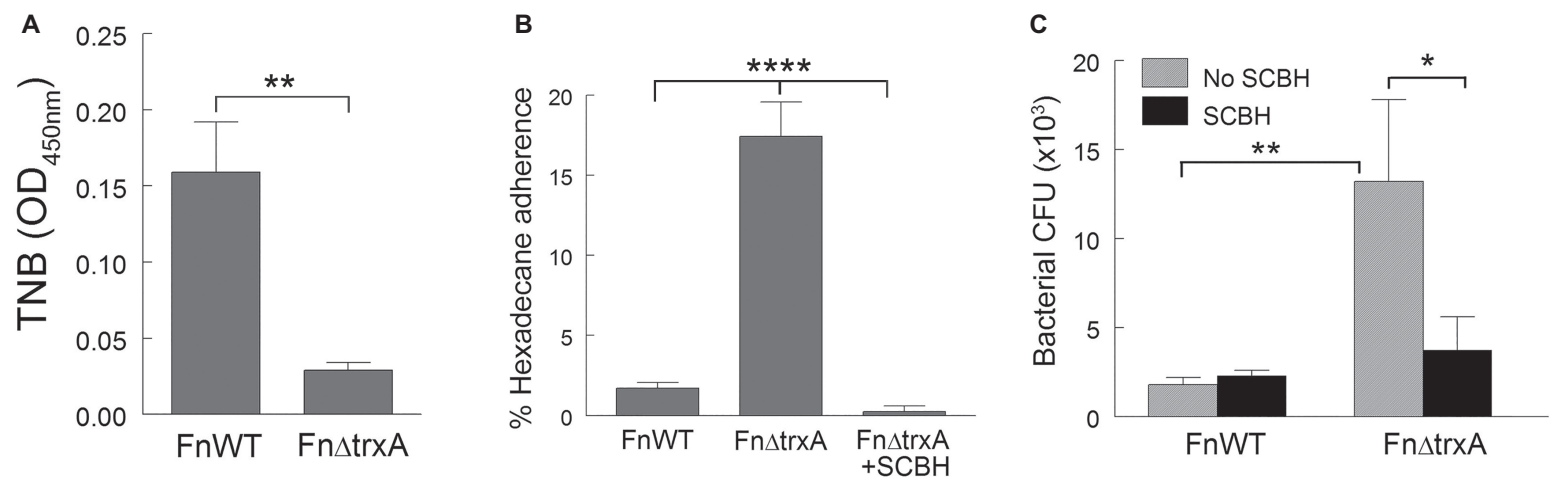
Thioredoxin modulates cell surface hydrophobicity in *Francisella novicida*. **(A)** Reduction of disulfide bonds by *F. novicida* wild type (FnWT) and FnΔtrxA measured through reduction of DTNB to TNB. **(B)** Adherence of Fn and FnΔtrxA to n-hexadecane with and without sodium cyanoborohydride (SCBH) treatment. **(C)** Recovered CFU/well of bacteria, with or without SCBH treatment, following phagocytosis by J774 cells. Statistical differences were determined by one-way **(A,B)** and two-way **(C)** ANOVA with Holm-Sidak correction for multiple comparisons (^****^p < 0.0001; ^**^p < 0.01; ^*^p < 0.05).

We further investigated whether CSH alteration by TrxA may be common to other Gram-negative bacteria by using *Francisella novicida*, which has a sequence-defined mutant library readily available (Gallagher et al., 2007). *F. novicida*, (a facultative intracellular pathogen) whose T4P biogenesis has been well studied, is closely related to the highly virulent human pathogen *Francisella tularensis* which was developed as a bioweapon (Dennis et al., 2001). First, the transposon mediated *F. novicida trxA* mutant (FnΔtrxA) was deficient in disulfide bond reduction as it was unable to convert DTNB to TNB to the extent of the WT U112 (FnWT) strain (Figure 4A), similar to *A. baumannii ΔtrxA* (Figure 2B). Concurrently, surface hydrophobicity of FnΔtrxA was significantly increased compared to the WT, but greatly reduced by SCBH treatment (Figure 4B) mirroring the pattern seen in *A. baumannii* (Figures 1B, 2C). When J774 cells were infected with *F. novicida* strains, the FnΔtrxA mutant was phagocytosed more readily than the FnWT, and the difference in bacterial uptake was abrogated with treatment by SCBH (Figure 4C), again replicating what was observed for *A. baumannii*. These data support the hypothesis that TrxA in other Gram-negative bacteria, in this case *F. novicida*, may modulate CSH and contribute to the interaction with host phagocytic cells.

### Bacterial Surface Hydrophobicity Is Associated With Type IV Pili Modulated by TrxA

**Figure 5:**
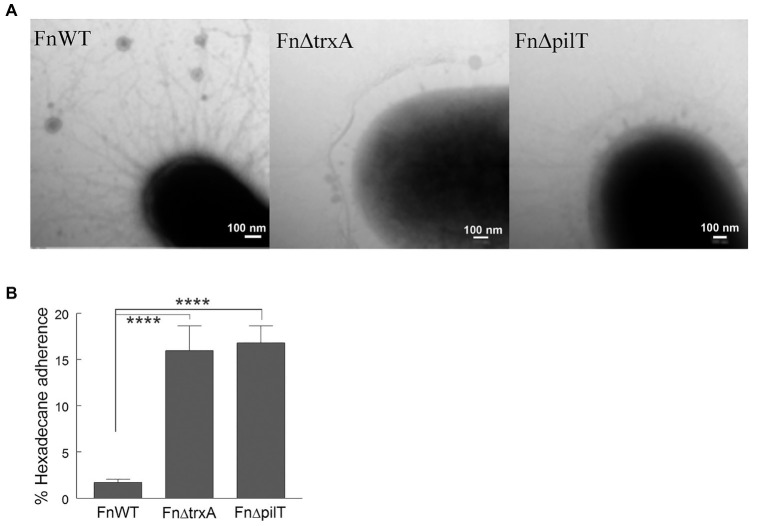
Changes in cell surface hydrophobicity are associated with lack of type IV pili. **(A)** Negatively-stained transmission electron microscopy images of *Francisella novicida* wild type (FnWT), FnΔtrxA, and FnΔpilT. **(B)** Adherence to n-hexadecane by Fn, FnΔtrxA, and FnΔpilT. Statistical differences were determined by one-way ANOVA with Holm-Sidak correction for multiple comparisons (^****^p < 0.0001).

Previously, we determined that *A. baumannii* ΔtrxA had a major T4P deficiency phenotype by RNA-seq analysis and transmission electron microscope (TEM) visualization (May et al., 2019). This finding prompted us to investigate the FnΔtrxA surface morphology under TEM. Similar to *A. baumannii* ΔtrxA, mutation of *trxA* in *F. novicida* had a profound effect on T4P. As shown in Figure 5A, reduction of pilus formation is evident in FnΔtrxA compared to FnWT and to an extent similar to the FnΔpilT bacterium. Because of the morphologic similarity between FnΔtrxA and FnΔpilT, we assessed whether FnΔpilT was also more hydrophobic than FnWT. Indeed, we observed a significant increase of hydrophobicity in FnΔpilT (Figure 5B), compared to FnWT, suggesting a second bacterial CSH modulation mechanism by TrxA which is mediated through alteration of T4P.

## Discussion

*Acinetobacter baumannii* has become a raising global health threat, but research into how *A. baumannii* maintains pathogenicity and virulence is still lacking. Previously, we demonstrated that *A. baumannii* ∆trxA is attenuated in virulence for mouse pathogenesis using gastrointestinal colonization (Ketter et al., 2018), systemic sepsis (Ainsworth et al., 2017), and pulmonary challenge (May et al., 2019) models. Transcriptome assessment by RNA-seq revealed global regulation of gene expression by TrxA (Gene Expression Omnibus accession number GSE125017) (May et al., 2019). TrxA has not been shown to be a transcription factor and TrxA-mediated gene regulation may be a secondary effect by modulation of multiple transcription factors in *A. baumannii*, as has been proposed before in experiments with *Bacillus subtilis* and *Rhodobacter* species (Li et al., 2004; Smits et al., 2005). Similarly, Cheng and colleagues suggest that TrxA is vital for protein folding and function by maintaining a highly reduced environment in the cytosol of *Listeria monocytogenes* (Cheng et al., 2017). They found that TrxA is essential for proper function of key regulators, including MogR, a transcriptional repressor which modulates flagella formation, and PrfA, a transcription factor which regulates several *Listeria* major virulence factors, such as ActA, Hpt, and LLO (Cheng et al., 2017). These studies demonstrated the complexity of TrxA-dependent cellular functions in bacterial pathogenesis.

We are reporting here a novel virulence mechanism by which bacteria modulate CSH *via* TrxA to evade the first line of host immune cells (e.g., phagocytes such as macrophages). This is likely a common mechanism shared by most *A. baumannii* isolates and other Gram-negative bacteria. Although only one *A. baumannii* strain (Ci79) strain was used in this study, the genetically distanced *F. novicida* did exhibit a similar TrxA phenotype to Ci79 in this study. It is also noted that TrxA protein sequence is identical among many *A. baumannii* strains and ΔtrxA of AB5075 and Ci79 shares a similar twitching mobility reduction phenotype. Two mechanisms of TrxA-mediated modulation of bacterial surface hydrophobicity identified in this study are disulfide bond reduction and regulation of T4P. How TrxA-mediated disulfide bond reduction can modulate CSH is not known. However, Penalver et al. reported that β-mercaptoethanol treatment released many cell wall proteins that may be responsible for the highly hydrophobic nature of *Aspergillus fumigatus* mycelia (Penalver et al., 1996). We have previously reported that *A. baumannii* TrxA dissociated secretory component from sIgA by disulfide bond reduction similar to β-mercaptoethanol treatment (Ketter et al., 2018). Whether *A. baumannii* secretes TrxA to reduce and/or release cell surface hydrophobic proteins remains to be elucidated.

We have used SCBH-treated bacteria in an *in vitro* J774 cell phagocytosis assay to investigate the possible role of bacterial surface hydrophobicity in TrxA-mediated immune evasion. SCBH is known to reduce imines to amines, but consensus is mixed on its ability to reduce disulfide bounds (Gidley and Sanders, 1982). It should be noted that the prevailing source for stating that SCBH does not reduce disulfide bonds (Jentoft and Dearborn, 1979) used 10 mM SCBH for 2 h, while this study used close to 30 mM for longer time frames. However, application of SCBH bacterial treatment to study the role of bacterial hydrophobicity in pathogenesis *in vivo* has its limitation. Although SCBH treatment had little impact on bacterial growth, the CSH modulation by SCBH could not be maintained beyond 3.5 h with cell surface dilution by replication.

The effects of T4P on hydrophobicity are not well explored in the literature. Previous work analyzing a *PilT* mutant in the *F. tularensis* LVS strain found that virulence was attenuated in a murine intradermal model in addition to a decreased ability of the mutant to adhere to macrophages, pneumocytes, and hepatocytes (Chakraborty et al., 2008). Beaussart et al. found that *P. aeruginosa* T4P bound strongly to hydrophobic surfaces. However, they also noted the complexity of these interactions as the flagella and PilY1 proteins had no effect on adherence (Beaussart et al., 2014). In *A. baumannii*, the T4P systems have been shown to have distinct glycoprotein diversity unrelated to the overall taxonomy of the organism and the T4P increased adhesion to epithelial cells, but did not affect biofilm formation (Piepenbrink et al., 2016). This same study also noted that a *P. aeruginosa* mutant lacking T4P was more susceptible to host opsonization and phagocytosis by immune cells. Overall, many questions remain on elucidating the complex interactions between bacterial appendages, CSH, and bacterial virulence. However, the data shown in this study supports the idea that thioredoxin may be playing a role in maintaining the T4P and that the T4P may modulate CSH.

## Data Availability Statement

All datasets generated for this study are included in the article/supplementary material.

## Author Contributions

HM, J-JY, MG, AC, and BA conceived and designed the experiments. HM, J-JY, and SS performed the experiments. HM, J-JY, SS, MG, JC, and BA analyzed the data. JS and KK contributed reagents and bacterial strains. HM and J-JY wrote the manuscript. MG and BA revised the manuscript.

### Disclaimer

The opinions or assertions contained herein are the private views of the authors and are not to be construed as official or as reflecting the views of the U.S. Department of the Army or the U.S. Department of Defense.

### Conflict of Interest

The authors declare that the research was conducted in the absence of any commercial or financial relationships that could be construed as a potential conflict of interest.
